# A New Void Fraction Measurement Method for Gas-Liquid Two-Phase Flow in Small Channels

**DOI:** 10.3390/s16020159

**Published:** 2016-01-27

**Authors:** Huajun Li, Haifeng Ji, Zhiyao Huang, Baoliang Wang, Haiqing Li, Guohua Wu

**Affiliations:** 1 State Key Laboratory of Industrial Control Technology, College of Control Science and Engineering, Zhejiang University, Hangzhou 310027, China; lihuajun@zju.edu.cn (H.L.); zyhuang@iipc.zju.edu.cn (Z.H.); blwang@iipc.zju.edu.cn (B.W.); hqli@iipc.zju.edu.cn (H.L.); 2 Key Laboratory of Complex Systems Modeling and Simulation of Ministry of Education, Hangzhou Dianzi University, Hangzhou 310018, China; wugh@hdu.edu.cn

**Keywords:** gas-liquid two-phase flow, small channel, void fraction, flow pattern, photodiode array sensor, laser diode

## Abstract

Based on a laser diode, a 12 × 6 photodiode array sensor, and machine learning techniques, a new void fraction measurement method for gas-liquid two-phase flow in small channels is proposed. To overcome the influence of flow pattern on the void fraction measurement, the flow pattern of the two-phase flow is firstly identified by Fisher Discriminant Analysis (FDA). Then, according to the identification result, a relevant void fraction measurement model which is developed by Support Vector Machine (SVM) is selected to implement the void fraction measurement. A void fraction measurement system for the two-phase flow is developed and experiments are carried out in four different small channels. Four typical flow patterns (including bubble flow, slug flow, stratified flow and annular flow) are investigated. The experimental results show that the development of the measurement system is successful. The proposed void fraction measurement method is effective and the void fraction measurement accuracy is satisfactory. Compared with the conventional laser measurement systems using standard laser sources, the developed measurement system has the advantages of low cost and simple structure. Compared with the conventional void fraction measurement methods, the proposed method overcomes the influence of flow pattern on the void fraction measurement. This work also provides a good example of using low-cost laser diode as a competent replacement of the expensive standard laser source and hence implementing the parameter measurement of gas-liquid two-phase flow. The research results can be a useful reference for other researchers’ works.

## 1. Introduction

In the past decades, the studies and industrial applications of gas-liquid two-phase flow in small-channel systems, such as chemical reactors, heat exchangers, refrigeration processes and micro-evaporators *etc.*, have become a significant area [1,2,3]. Void fraction is an important parameter of the two-phase flow. Its on-line measurement is of great importance for the heat and mass transfer efficiency, the estimation of pressure drop, the process control and the measurement of other parameters of the two-phase flow [4,5,6,7,8,9]. However, with the decrease of the channel dimension, the measurement of the void fraction becomes more and more difficult. The conventional measurement methods cannot fulfill the growing requirements of the industrial applications and the mechanism studies of the two-phase flow [1,2,3,4,5,6,7,8,9].

Due to its advantages of high spatial and temporal resolution, the optical measurement technique is a very attractive and useful approach to implement the parameter measurement of gas-liquid two-phase flow in small channels [10,11,12]. The conventional optical measurement methods can be mainly divided into three categories: optical probe method, visualization method, and laser-based method [10,11,12]. Because the optical probe is directly in contact with the detected fluid, the optical probe method will more or less disturb the practical flow of the fluid. In addition, the probes may be contaminated and unpredictable measurement error will arise [13,14,15]. The visualization method includes high-speed cameras, digital cameras, and optical tomography *etc.* [16,17,18]. Although the measurement performance of the visualization method is satisfactory, the visualization method has the disadvantages of high cost, complicated construction and higher requirement of the measurement environment [16,17,18]. Many laser-based methods have been proposed and widely studied, including laser Doppler velocimetry, laser induced fluorescence and particle image velocimetry, *etc*. However, the conventional laser based methods need an expensive laser source (e.g., Nd–Yag laser source or He-Ne laser source.) and a complicated measurement system (including seeding particle, fluorescent particle and objective lens, *etc.*) [19,20,21]. These methods also have the disadvantages of high cost and higher requirements of the measurement environment. Therefore, although significant technical achievements and progresses have been obtained, due to the above mentioned disadvantages, more research works should be undertaken to seek a more effective optical method to implement the parameter measurement of gas-liquid two-phase flow in small channels with the advantages of lower cost, simpler construction and better capability of the environment [10,11,12].

Currently, the techniques of information science and micro-electronics have been rapidly developed. As a new photo-electric device, the performance of photodiode sensor has been significantly improved. The dimension of photodiode sensing element has been greatly decreased and a photodiode array sensor can be successfully developed with much lower cost/price [22,23]. As a new kind of laser source, in some cases, the laser diode can be used as a low-cost alternative of the conventional expensive laser source [24,25]. These technical progresses have laid a solid foundation of developing a low-cost optical measurement system. Meanwhile, as a newly emerging signal processing technology, machine learning, which aims to implement data mining, pattern recognition and modeling, *etc.*, has been widely applied and studied in many research fields. Machine learning technology can provide useful approaches to make full use of the measurement information and hence to implement the parameter measurement successfully [26,27,28,29]. However, up to date, our knowledge and experience on the applications of the above new devices and machine learning technology to the parameter measurement of gas-liquid two-phase flow in small channels are very limited [4,5,6,7,8,9].

Based on a photodiode array sensor and a laser diode, this work aims to develop a low-cost void fraction measurement system and hence to propose a new optical measurement method for the void fraction measurement of gas-liquid two-phase flow in small channels by using machine learning technology.

## 2. Void Fraction Measurement System and Measurement Scheme

**Figure 1:**
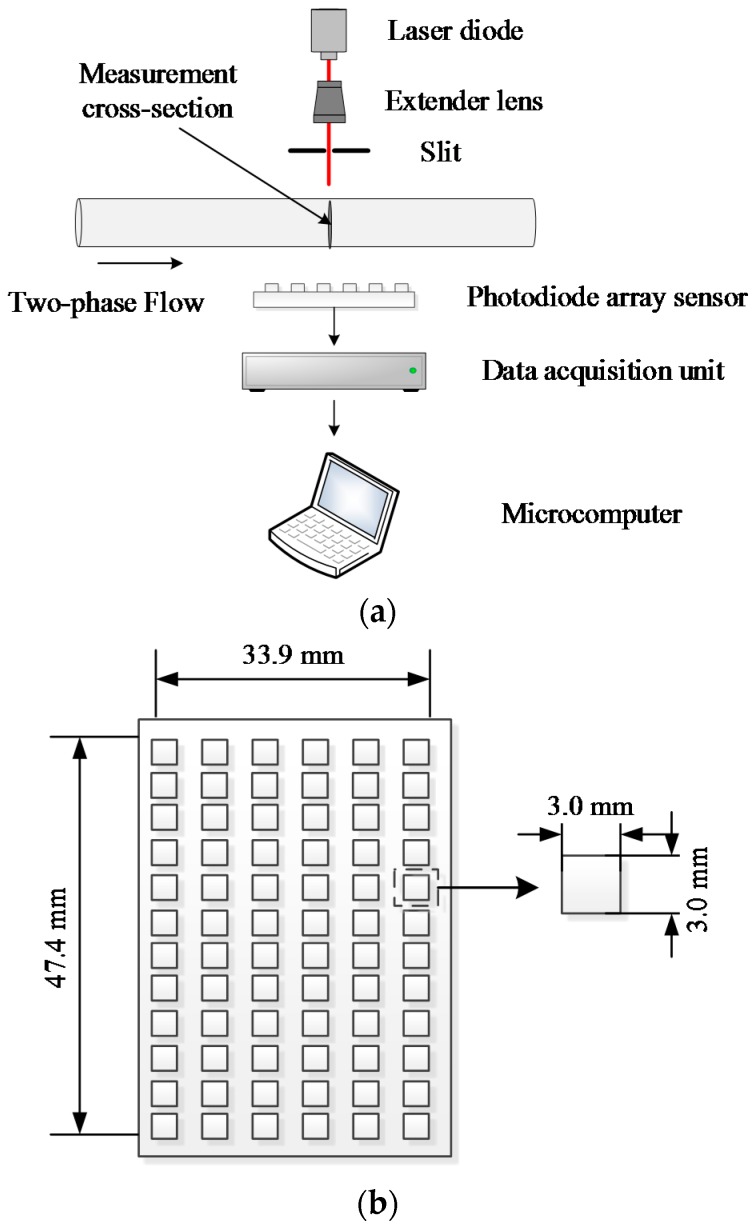
(**a**) The structure of the new void fraction optical measurement system; (**b**) The layout of the photodiode array sensor.

Figure 1a shows the structure of the new void fraction optical measurement system, including a laser diode, an extender lens, a slit, a photodiode array sensor, a data acquisition unit and a microcomputer. The laser diode which is used to produce a beam of laser is YuanDa laser L63510P5 with a wavelength of 635 nm (the wavelength of the laser diode is chosen according to our experience and previous experimental results) and an output power of 5 mW. The extender lens and the slit are used to change the laser into a parallel laser sheet (The extender is used to extend the beam diameter and decrease the laser’s divergence. In this work, the resulting laser through the extender lens has a diameter of 40 mm). The laser sheet passes through the transparent channel perpendicularly, and is absorbed, reflected or deflected by the gas-liquid two-phase flow inside the small channel. The exit laser, which contains the characteristic information of the two-phase flow, is recorded by the array sensor. The output signals of the array sensor are then transmitted to the microcomputer by the data acquisition unit. Figure 1b illustrates the layout of the photodiode array sensor. According to the required sensing area, the cost and the size of the sensing element, the array sensor consists of 72 (12 × 6) sensing elements. The outputs of the 72 sensing elements will be sent into the micropucter simultaneously. The sensing element is Vishay Telefunken PIN photodiode BPW34, which has a sensing area of 3.0 × 3.0 mm^2^ (in this work, the Signal to Noise Ratio of the sensing element is about 30 dB). Meanwhile, it is necessary to indicate that the number of the sensing elements is determined by our previous experimental results. It is not an optimal number. To look for an optimal element number of the array sensor will be our further research.

**Figure 2:**
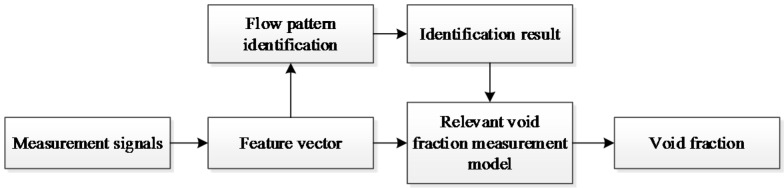
The scheme of the void fraction measurement.

Research works have verified that the flow patterns of gas-liquid two-phase flow have significant influences on the measurement of the void fraction [1,2,3,4,5,6,30]. If we use one measurement model for the void fraction measurment, the measurement accuracies will not be satisfactory. An effective approach to solve this problem is to develop different measurement models for different flow patterns. Thus, the real time flow pattern identification result is necessary and the identification result is introduced to the void fraction measurment. In this work, the real-time flow pattern identification result is introduced into the void fraction measurement process. Meanwhile, for each typical flow pattern, a specific void fraction measurement model is developed. Figure 2 shows the scheme of the void fraction measurement. With the obtained measurement signals (a total of 72 groups of optical signals obtained by the array sensor), a feature vector is firstly extracted. According to the feature vector, the real-time flow pattern identification is then implemented. Finally, according to the flow pattern identification result, a relevant void fraction measurement model is selected to calculate the void fraction.

To obtain the comprehensive information of the two-phase flow, the feature vector is extracted from the 72 measurement signals obtained by the photodiode array sensor (each measurement signal is obtained by one sensing element and contains a series of data points). The feature vector consists of two groups of statistical features which contain useful information of the gas-liquid two-phase flow, the mean values and the standard deviations of the 72 measurement signals. 

The mean value represents the time-averaged characteristic of a measurement signal. The mean value of the measurement signal obtained by the *k_th_* sensing element *m_k_* is

(1) $$m_{k}=\frac{1}{L}\overset{L}{\underset{i=1}{\sum }}u_{k}\left(i\right)$$

where *L* is the data length of the measurement signal, *u_k_* is the measurement signal obtained by the *k_th_* sensing element, and *k* = 1,2,3,…,72.

The standard deviation represents the dispersion of a measurement signal. The standard deviation of the measurement signal obtained by the *k_th_* sensing element *d_k_* is

(2) $$d_{k}=\sqrt{\frac{1}{L-1}\overset{L}{\underset{i=1}{\sum }}{\left(u_{k}\left(i\right)-m_{k}\right)}^{2}}$$

Thus, combining the two groups of statistical features, the feature vector *x* is obtained

(3) $$x={\left[m_{1},\ldots ,m_{72},d_{1},\ldots ,d_{72}\right]}^{T}$$

The flow pattern identification is a pattern recognition problem. The development of a void fraction measurement model is a modeling problem. As mentioned in Section 1, machine learning technology can provide many useful approaches to solve pattern recognition problems or modeling problems, such as *k*-nearest neighbor, linear discriminant analysis, and Bayes classifier, *etc.* can implement the pattern recognition [26,27,28,29], while linear regression, artificial neural network and Bayesian learning, *etc.* can implement the modeling [26,27,28,29].

Compared with the other pattern recognition techniques mentioned above, Fisher Discriminant Analysis (FDA) is a dimensionality reduction technique and can provide a linear transformation that maximizes the between-class scatter and minimizes the within-class scatter. FDA has been widely used in the pattern identification field, and its effectiveness has been verified [26,27,28,29,31,32]. Thus, in this work, FDA is adopted to implement the flow pattern identification. Compared with the other modeling techniques mentioned above, Support Vector Machine (SVM) is a valid machine-learning technique and is suitable for model developing in small sample conditions. SVM has good generalization ability and has been widely used in many fields for model development [26,27,28,29,33]. Therefore, in this work, SVM is selected to develop the void fraction measurement models.

## 3. Flow Pattern Identification

**Figure 3:**
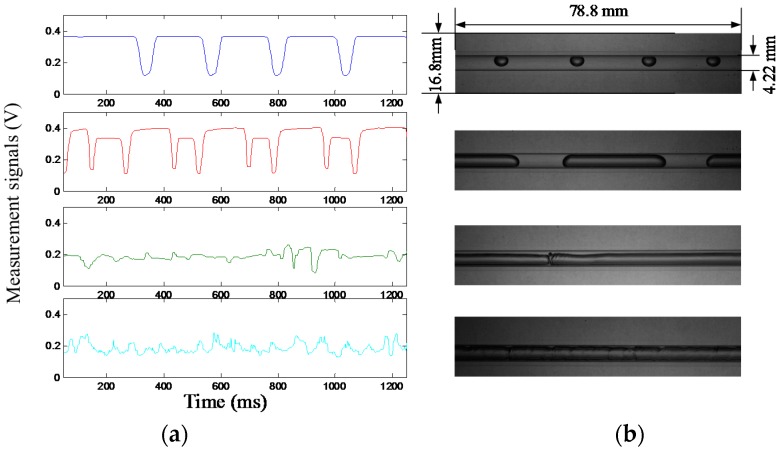
(**a**) Measurement signals of the four flow patterns obtained by one sensing element; (**b**) Images of the four flow patterns obtained by a high-speed camera.

Four typical flow patterns of gas-liquid two-phase flow in small channels are investigated in this work, including bubble flow, slug flow, stratified flow, and annular flow. According to our experimental results, the measurement signals of the bubble flow and the slug flow have some similarities, while the measurement signals of the stratified flow and the annular flow have some similarities. Figure 3 shows typical groups of the measurement signals and the images of the four flow patterns. The measurement signals are obtained by a sensing element (*i.e.*, the sixth BPW34 diode at the fourth column of the photodiode array sensor). The images of the flow patterns are obtained by a high-speed camera (Intergrated Design Tools, Inc. (IDT) Redlake MotionXtra N-4).

**Figure 4:**
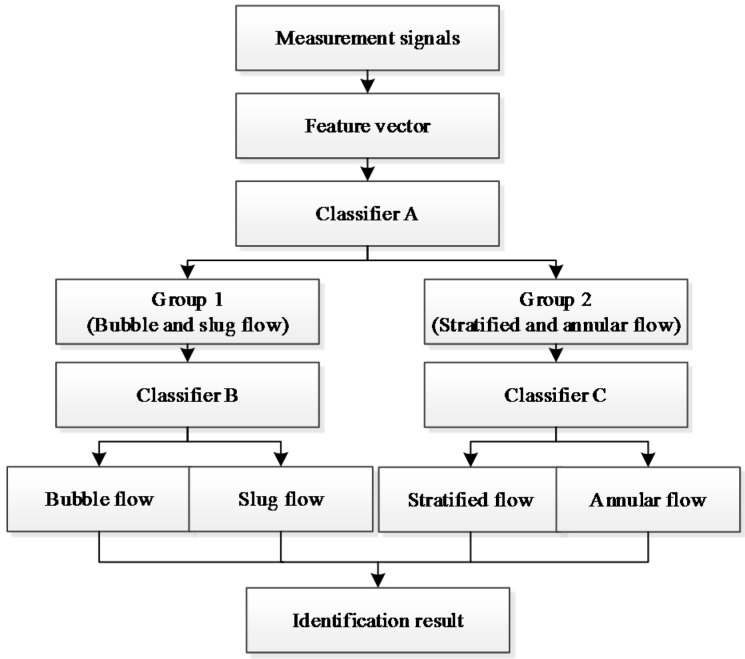
The flowchart of the flow pattern identification.

As shown in Figure 3, in the bubble flow, when a gas bubble passes through the measurement cross-section, the measurement signal has a clear voltage decrease and stays steady when the channel is full of water. In the slug flow, the measurement signal also has clear voltage decrease when a gas slug approaches and leaves, while at the central part of the gas slug, the measurement signal remains invariable. The measurement signals of the bubble flow and the slug flow have some similarities, but the voltage decrease amplitudes of the signals are different. In the annular flow or the stratified flow, the measurement signals both display fluctuations. However, the amplitude of the measurement signal fluctuation in the annular flow is different from that in the stratified flow. Therefore, based on the above characteristics of the measurement signals in different flow patterns, in this work, the bubble flow and the slug flow are initially classified as one group (Group 1), and the stratified flow and the annular flow are initially classified as the other group (Group 2). Figure 4 shows the flowchart of the flow pattern identification. The process of the flow pattern identification has two key steps. The first step is to determine that the real-time flow pattern belongs to Group 1 or Group 2 by Classifier A. The second step is to determine the specific real-time flow pattern by Classifier B or C.

The three classifiers (Classifier A, B, and C) are developed by FDA and each classifier is aimed to solve a binary classification problem. The decision function of the binary classifier can be expressed as:

(4) $$y\left(x\right)=\text{sign}\left[\omega^{T}x+\omega_{0}\right]$$

where *y* is the class label (*y* = −1 or 1), *x* is the feature vector, *ω* is the Fisher vector and *ω_0_* is the threshold. *ω* can be determined by solving the following problem:

(5) $$J\left(\omega \right)=\text{max}\frac{\omega^{T}S_{b}\omega }{\omega^{T}S_{w}\omega }$$

where *S_w_* is the within-class-scatter matrix and *S_b_* is the between-class-scatter matrix. Once the classifier is developed, by inputting the feature vector *x* into the decision function, the identification result can be obtained according to the class label *y*. The detailed description concerning FDA is available in [26,27,28,29].

## 4. Void Fraction Measurement Model Development

**Figure 5:**
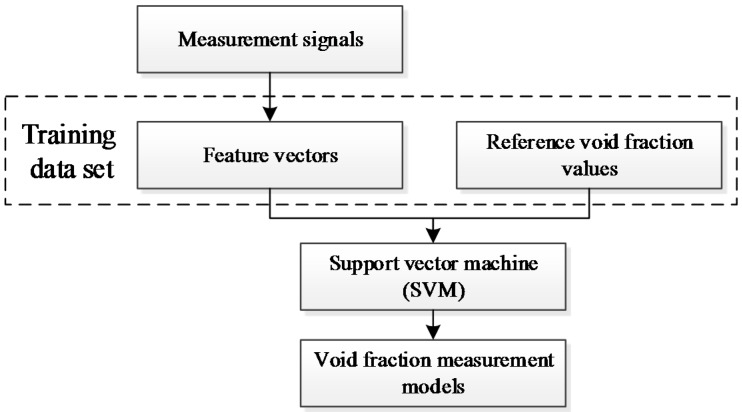
The flowchart of the void fraction measurement model development.

Figure 5 shows the flowchart of the void fraction measurement model development. For each typical flow pattern, a specific void fraction measurement model is developed. According to experimental results, a training data set is constructed. The training data set includes the sample feature vectors extracted from the experimental measurement signals and the reference void fraction values. With the training data set, the void fraction measurement models of the four flow patterns (totally four models) are developed by SVM.

According to the principle of SVM, the void fraction measurement model can be expressed as:

(6) $$\alpha \left(x\right)=\overset{p}{\underset{i=1}{\sum }}\left(\beta_{i}-\beta_{i}^{*}\right)K\left(x,x_{i}\right)+b$$

where *α* is the void fraction, *x* is the feature vector, and ${\left\{x_{i,\;}\alpha_{i}\right\}}_{i=1}^{p}$ is the training data set with *p* samples. *K(x,x_i_)* is kernel function. In this work, the radial basis function $K\left(x,x_{i}\right)=\exp\left(-{\left|x-x_{i}\right|}^{2}\right)/\sigma^{2}$ is selected as the kernel function, and *σ* is its diameter. *b* is a parameter. *β_i_* and β_i_* are the Lagrange multipliers, which are determined by solving the following optimization problem:

(7) $$\left\{ \begin{array}{c} min\left[\frac{1}{2}\overset{p}{\underset{i=1,j=1}{\sum }}\left(\beta_{i}-\beta_{i}^{*}\right)\left(\beta_{j}-\beta_{j}^{*}\right)K\left(x_{i},x_{j}\right)-\overset{p}{\underset{i=1}{\sum }}\left(\beta_{i}-\beta_{i}^{*}\right)\alpha_{i}+\overset{p}{\underset{i=1}{\sum }}\left(\beta_{i}+\beta_{i}^{*}\right)\varepsilon \right]\\ s.t.\;\{\begin{array}{c} 0\leq \beta_{i},\beta_{i}{}^{*}\leq C,i=1,\ldots ,p\\ \overset{p}{\underset{i=1}{\sum }}\left(\beta_{i}-\beta_{i}^{*}\right)=0,i=1,\ldots ,p \end{array} \end{array}\right.$$

where *ε* is the slack variable and *C* is the penalty factor. The detailed description concerning the SVM is available in [27,28,29].

## 5. Practical Process of the Void Fraction Measurement

**Figure 6:**
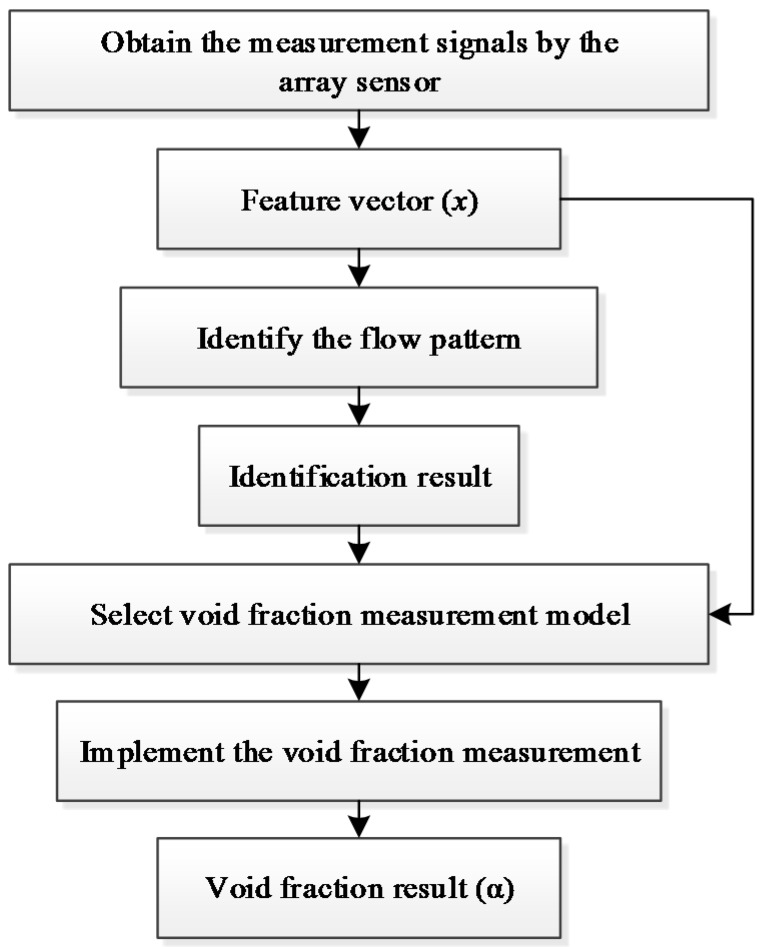
The practical process of the void fraction measurment.

The practical process of the void fraction measurement is illustrated in Figure 6. Firstly, the 72 measurement signals of the gas-liquid two-phase flow are obtained by the array sensor, and the feature vector *x* of the signals is extracted. Secondly, the real-time flow pattern is identified. Then, according to the identification result, a relevant void fraction measurement model is selected. Finally, with the selected measurement model and the obtained feature vector *x*, the void fraction measurement *α* is obtained.

## 6. Experimental Setup

**Figure 7:**
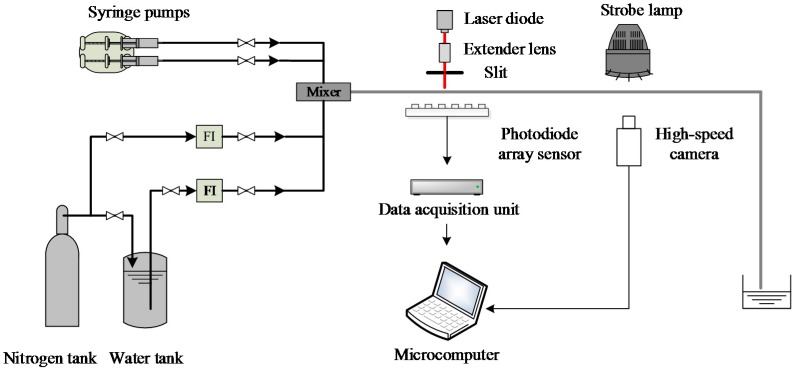
Experimental setup of the void fraction measurement system for gas-liquid two-phase flow in small channels.

Figure 7 shows the experimental setup of the void fraction measurement system for gas-liquid two-phase flow in small channels. The gas and the liquid phase are driven into the small channel by syringe pumps or a nitrogen tank (if either the gas flowrate or the liquid flowrate is less than 3.6 L/h, the corresponding syringe pump is used; otherwise, the nitrogen tank is used). Nitrogen is used as the gas phase and its flowrate ranges from 0 to 1300 L/h. Tap water is used as the liquid phase, and its flowrate ranges from 0 to 20 L/h. The two phases mix at the mixer, and then the two-phase flow flows through a horizontal channel with a length of 0.95 m. The distance between the channel inlet and the photodiode array sensor is 0.25 m. The optical measurement signals of the two-phase flow are obtained by the photodiode array sensor and then sent to the microcomputer by the data acquisition unit. Meanwhile, an IDT Redlake MotionXtra N-4 high-speed camera (maximum fps (frames per second) @ full resolution: 3000 fps @ 1024 × 1024) is used to obtain the images of the flow patterns.

Four small channels with inner diameters (i.d.) of 4.22, 3.04, 2.16 and 1.08 mm, respectively, are used in the experiments. Four typical flow patterns including bubble flow, slug flow, stratified flow and annular flow are investigated. The sampling frequency of the photodiode array sensor is set to 1 kHz. The National Instruments cDAQ-9172 is selected as the data acquisition unit. The reference data of the void fraction is determined by the quick-closing valve method [4,5,6].

## 7. Experimental Results and Discussions

### 7.1. Experimental Results

**Figure 8:**
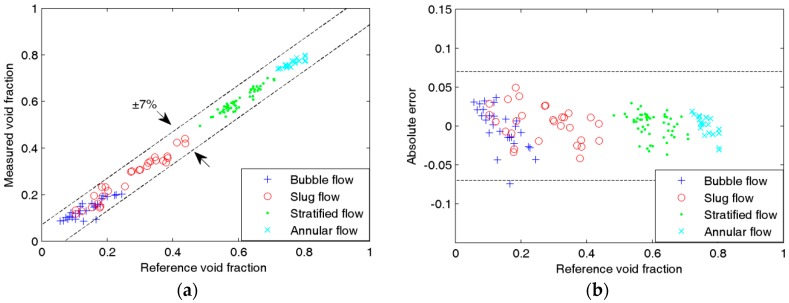
Experimental results of the void fraction measurement in the 4.22-mm i.d. channel: (**a**) Comparison between measured and reference void fractions; (**b**) Absolute errors of the void fraction measurement.

**Figure 9:**
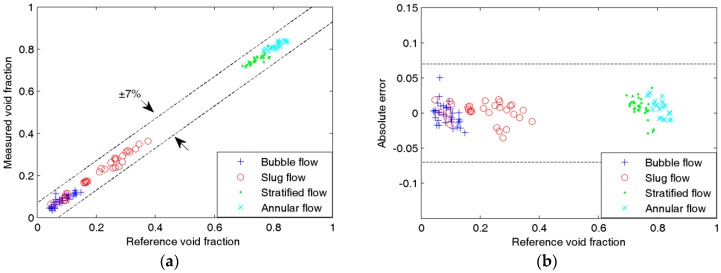
Experimental results of the void fraction measurement in the 3.04-mm i.d. chanel: (**a**) Comparison between measured and reference void fractions; (**b**) Absolute errors of the void fraction measurement.

**Figure 10:**
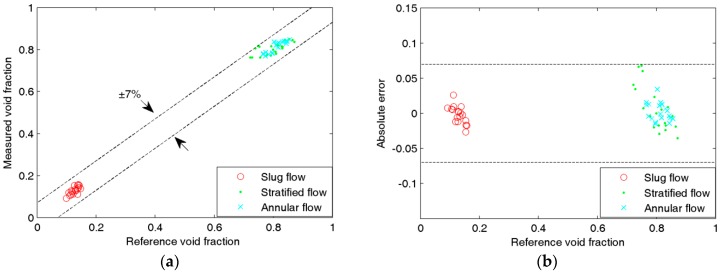
Experimental results of the void fraction measurement in the 2.16-mm i.d. chanel: (**a**) Comparison between measured and reference void fractions; (**b**) Absolute errors of the void fraction measurement.

**Figure 11:**
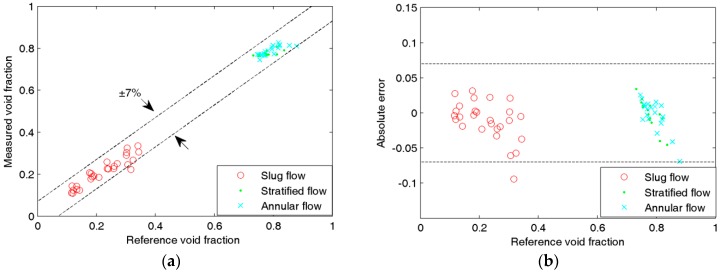
Experimental results of the void fraction measurement in the 1.08-mm i.d. chanel: (**a**) Comparison between measured and reference void fractions; (**b**) Absolute errors of the void fraction measurement.

Figure 8, Figure 9, Figure 10 and Figure 11 show the experimental results of the void fraction measurement in the four small channels. Compared with the reference void fractions obtained by the quick-closing valve method, the maximum absolute errors of the void fraction measurement in the four small channels are all less than 7%.

The experimental results indicate that the development of the void fraction measurement system is successful and the proposed void fraction measurement method is effective.

### 7.2. Discussion

In this work, a photodiode array sensor is used to obtain the signals of the exit laser. With 72 sensing elements, the array sensor has enough sensing area to obtain sufficient signals of the exit laser. Then, comprehensive information of the two-phase flow can be acquired from the obtained signals, and the void fraction measurement can be implemented. Meanwhile, in the proposed measurement method, a low-cost laser diode is used as the laser source. According to the current technique level, the performance indexes (such as output power, laser coherence and divergence, *etc.*) of the conventional standard laser sources (e.g., Nd: YAG laser source, He-Ne laser source, *etc.*) are comprehensively better than that of the laser diode. The only comparable performance index of the laser diode is the power stability (Power stability is the maximum drift with respect to mean power over eight hours. In this work, the laser diode has a power stability of 2%, while the standard laser sources such as THORLABS HNL050L He-Ne laser source have a power stability of 2.5% [34]). The proposed measurement method is mainly on the basis of the power changes and distribution of the exit laser. The power stability is the key performance index of the laser source. Thus, in this work, the advantage of the laser diode in power stability is fully utilized. Furthermore, with the supports of suitable machine learning techniques and the developed photodiode array sensor, the low-cost laser diode successfully acts as a competent replacement of the expensive standard laser source. Sufficient information concerning the characteristic of the two-phase flow is obtained and processed. Finally, the void fraction measurement is implemented.

To implement the void fraction measurement with a satisfactory accuracy, the influence of the flow pattern should be condersided. Compared with the normal scale channel, the flow characteristics of the two-phase flow in small channels have significant differences. As the dimension of the channel decreases, some flow patterns become common and the others are difficult to observe [2,3,4,5]. According to the experimental results, bubble flow is observed in the 4.22-mm and 3.04-mm i.d. channels but not in the 2.16-mm and 1.08-mm i.d. channels, while the slug flow, stratified flow and annular flow are all observed and investigated in the four small channels. These experimental results may provide useful reference for others’ research work.

To overcome the influence of the flow pattern on the void fraction measurement, a void fraction measurement method is proposed. In this method, a specific void fraction measurement model is developed for each typical flow pattern. In practical measurement, the parameters of the models vary with the flow patterns, which means that the flow pattern indeed has significant influence on the void fraction measurment. To overcome the influence of the flow pattern, the flow pattern of the two-phase flow is identified at first, and then, according to the identification result, a relevant void fraction measurement model is selected to implement the void fraction measurement. The experimental results show that, with the introduction of the real-time flow pattern identification result, the influence of the flow pattern on the void fraction measurement has been significantly reduced.

## 8. Conclusions

In this work, based on a photodiode array sensor and a laser diode, a low-cost and simple-structure void fraction measurement system for gas-liquid two-phase flow in small channels is developed. A low-cost laser diode is adopted as the laser source and a 12 × 6 photodiode array sensor is used to obtain the information concerning the two-phase flow. Meanwhile, a new void fraction measurement method is proposed. The machine learning techniques (FDA and SVM) are adopted to implement the flow pattern identification and the development of the void fraction models. To overcome the influence of flow pattern on the void fraction measurement, the identification result is introduced to the void fraction measurement. Then, according to the identification result, a relevant void fraction measurement model is selected to implement the void fraction measurement.

Experiments are carried out in four small channels with different inner diameters of 4.22, 3.04, 2.16 and 1.08-mm, respectively. Four typical flow patterns including bubble flow, slug flow, stratified flow and annular flow are investigated. The maximum absolute error of the void fraction measurement is less than 7%. The experimental results show that the proposed void fraction measurement method is effective and the development of the measurement system is successful. The experimental results also show that the introduction of the flow pattern information can overcome the influence of the flow pattern on the void fraction measurement.

The research results also verify that the low-cost laser diode can act as a competent replacement of the expensive standard laser source if suitable signal processing techniques and information acquisition techniques are used. That can significantly reduce the cost of the laser based measurement system. This research work can provide a good reference for other researchers’ works.
